# Associations between Nausea, Vomiting, Fatigue and Health-Related Quality of Life of Women in Early Pregnancy: The Generation R Study

**DOI:** 10.1371/journal.pone.0166133

**Published:** 2016-11-04

**Authors:** Guannan Bai, Ida J. Korfage, Esther Hafkamp-de Groen, Vincent W. V. Jaddoe, Eva Mautner, Hein Raat

**Affiliations:** 1 Department of Public Health, Erasmus MC, University Medical Center Rotterdam, Rotterdam, the Netherlands; 2 The Generation R Group, Erasmus MC, University Medical Center Rotterdam, Rotterdam, the Netherlands; 3 Department of Epidemiology, Erasmus MC, University Medical Center Rotterdam, Rotterdam, the Netherlands; 4 Department of Obstetrics and Gynaecology, Medical University of Graz, Graz, Austria; Universite de Rennes 1, FRANCE

## Abstract

The objective of this study was to evaluate the independent associations between nausea, vomiting, fatigue and health-related quality of life of women in early pregnancy in the Generation R study, which is a prospective mother and child cohort. Analyses were based on 5079 women in early pregnancy in the Rotterdam area, the Netherlands. The information on nausea, vomiting and fatigue in the previous three months was measured in the questionnaire at enrollment, as well as potential confounders (i.e., maternal/gestational age, ethnic background, educational level, parity, marital status, body mass index, tobacco and alcohol use, chronic/infectious conditions, uro-genital conditions/symptoms, sleep quality, headache, anxiety, and depression). Health-related quality of life was assessed by the 12-item Short Form Health Survey and physical and mental component summary scores were calculated. Multivariate regression models were performed to evaluate the independent associations of the presence of nausea, vomiting and fatigue with health-related quality of life, adjusting for potential confounders. 33.6% of women experienced daily presence of nausea, 9.6% for vomiting and 44.4% for fatigue. Comparing with women who never reported nausea, vomiting and fatigue, women with daily presence of at least one of these symptoms had significantly lower scores of physical component summary and mental component summary, after adjusting for potential confounders. Our study shows how common nausea, vomiting and fatigue are among women in early pregnancy and how much each of these symptoms negatively impact on health-related quality of life. We call for awareness of this issue from health care professionals, pregnant women and their families.

## Introduction

Nausea, vomiting and fatigue are the most common symptoms in early pregnancy; more than 70% of women have reported the presence of these symptoms in previous studies [1–3]. Causes of nausea, vomiting and fatigue during pregnancy remains unknown; rising levels of hormone and stress might be risk factors [4, 5]. Typically, nausea and vomiting begin around gestational weeks 5–8 with peak symptoms occurring around gestational weeks 9 and subsiding around week 12 [6, 7]. Some studies show that fatigue increases over time throughout the whole pregnancy; other studies indicate that fatigue in the first trimester is worse than in the third trimester [8–10].

Nausea, vomiting and fatigue may affect the physiological, psychological and emotional aspects of women’s lives, and may diminish women’s quality of life (QOL) [3, 9, 11, 12]. QOL reflects subjective perceptions of the individual's position in life in the context of the culture and value systems in which he or she lives, and in relation to the individual's goals, expectations, and concerns [13]. QOL refers to holistic well-being, whereas health-related quality of life (HRQOL) focuses on health-related aspects of well-being [14]. Recently, an increasing attention has been paid to associations between pregnancy-related symptoms and HRQOL [15–23]. Some studies have indicated the relatively low score for many domains of HRQOL among women with presence of nausea and vomiting [15–20, 22, 23], for instance considering the 36 item Short Form Health Survey (SF-36) subscale scores on physical functioning (61.1 vs. 88.9), vitality (23.2 vs. 62.8) and social functioning (44.7 vs. 84.6) in comparison with the general population women aged 14–44 years [20]. SF-36 is an often-used generic QOL measure. Lacasse et al. showed that the presence of nausea and vomiting of pregnancy in the first trimester was significantly associated with lower scores considering the 12 item Short Form Health Survey (SF-12) physical component summary scale (p<0.0001) and mental component summary score (p = 0.0066) [17]. SF-12 closely mirrors the SF-36 with a good reliability and validity [24]. In two other studies, a negative association with the physical domain of HRQOL was observed [21, 25]. The inconsistent findings may be due to differences in study design and the timing and mode of measurements, or it may be due to the small sample sizes. Little evidence is available regarding the HRQOL of women in early pregnancy in community samples. Data on associations between fatigue and HRQOL is scarce. Few studies applied multivariate regression models [17, 18], and many of the previous studies employed bivariate analysis [20–22].

In the present study, we present data of 5079 mothers participating in a population-based prospective mother and child cohort in the Netherlands. We aimed to evaluate the independent associations of nausea, vomiting and fatigue with HRQOL of women in early pregnancy.

## Methods

### Data Source

This study was embedded within the Generation R study, a population-based prospective mother and child cohort study, designed to identify early environmental and genetic causes of normal and abnormal growth, development and health from fetal life until young adulthood. The Generation R study has been previously described in detail [26–29]. In total, 9778 mothers with a delivery date from April 2002 until January 2006 were enrolled in pregnancy (n = 8879) or at birth of their children (n = 899) in the entire Generation R Study. This includes 7069 women, who were enrolled in early pregnancy (<18 weeks of gestation, median: 13 weeks). The overall response rate of the study was 61% [29]. The assessments in prenatal phase were conducted using three questionnaires: Mother 1 Questionnaire in early pregnancy; Mother 3 Questionnaire in mid-pregnancy (18–25 weeks of gestation); Mother 4 Questionnaire in late pregnancy (gestational age ≥25 weeks) [27]. Overall, mothers received four postal questionnaires during the prenatal phase; the three questionnaires that were just mentioned above plus Mother 2 Questionnaire regarding diet. The 25-page Mother 1 Questionnaire was used for the present study and assessed at around 12 weeks of gestation. It includes topics of medical history, family history, previous and current pregnancies, quality of life, life style habits, housing conditions, ethnicity and educational level [27]. The study was conducted with the guideline proposed in the World Medical Association of Helsinki and has been approved by the Medical Ethical Committee of the Erasmus Medical Center, University Medical Center Rotterdam. Written consent was obtained from all of the participating women [30].

### Study population

Seven thousand and sixty-nine women were enrolled before 18 weeks of their gestation [26]. The assessment by Mother 1 Questionnaire was planned at around 12 weeks of their pregnancy (median: 13 weeks). We excluded women who didn’t respond to the questionnaire (n = 497). Additionally, we excluded pregnancies with the following outcomes: twin pregnancies (n = 71), induced abortion (n = 23), fetal deaths before 20 weeks of gestation (n = 62), loss to follow up their pregnancy outcomes (n = 23). Further, we excluded women with missing data on the symptoms (nausea, vomiting and fatigue) (n = 158). Finally, we excluded women in case of lacking information on one or more items of the SF-12 (n = 1156). Thus, the population for analysis comprised 5079 pregnant women (see S1 Fig).

### Measurement of symptoms

The questions posed to pregnant women regarding to nausea, vomiting and fatigue are ‘have you had nausea in the last three months’, ‘have you had vomiting in the last three months’ and ‘have you had tiredness in the last three months’. The possible responses were ‘daily, a few days a week, once per week, less than once per week and never’. ‘The last three months’ refers the latest three months before the subject completed the questionnaire. By using ‘never’ as the reference group, the other four categories were recoded as dummy variables for multiple regression analyses.

### Health-related quality of life

Women’s HRQOL in the past month was measured by SF-12 in the questionnaire at around 12 weeks of gestation (median: 13 weeks). SF-12 yields two component summaries: the physical component summary (PCS) and the mental component summary (MCS) [24, 31]. The Cronbach’s alpha for SF-12 in our study is 0.83. SF-12 includes 12 items regarding 8 scales: physical functioning (two items), role limitations due to physical problems (two items), bodily pain (one item), general health (one item), vitality (one item), social functioning (one item), role limitation due to emotional problems (two items) and perceived mental health (two items). Recoding for some items was conducted, so that a high value indicated the same type of response for each item. Then the raw scores were transformed to provide scale scores that ranged from 0 (the worst) and 100 (the best). We then calculated the raw physical component summary score and the raw mental component summary score by summing up all the scale scores weighted based on US general population survey. Finally the raw PCS and MCS scores were transformed into the standard scores based on the normalized algorithms from the US general population with the mean value of 50 (add 50) and the standard deviation of 10 (multiply by 10) [31]. The standardization enables cross-cultural comparison [32].

### Covariates

Based on previous studies of determinants of pregnant women’s HRQOL, we considered the demographic characteristics, life-style related factors, and indicators of health status as potential confounders [9, 17, 18, 33, 34]. Data on these variables were collected in self-reported questionnaires at enrollment. The demographic characteristics included maternal age, gestational age, ethnic background (native Dutch people, other Western immigrant and non-Western immigrant), educational level (low, mid-low, mid-high, high), parity, marital status. Maternal ethnic background and education level were defined according to the classification of Statistics Netherlands [35]. Education was categorized into four subsequent levels based on the Dutch Standard Classification of Education: high (university degree), mid-high (higher vocational training, Bachelor’s degree), mid-low (>3 years general secondary school, intermediate vocational training) and low (no education, primary school, lower vocational training, intermediate general school, or 3 years or less general secondary school) [36].

Lifestyle-related factors included body mass index (BMI), tobacco and alcohol use; indicators of health status included chronic non-infectious conditions, infectious/inflammatory conditions, uro-genital symptoms, sleep quality, headache, anxiety, and depression. Tobacco/alcohol use was measured by asking ‘have you smoked in the past three months” and “have you drunk any alcohol in the past three months”, respectively. The amount of alcohol use was also measured.

Women were asked whether in the past 12 months they had one or more of 14 chronic non-infectious conditions on the standard list of chronic conditions according to Statistics Netherlands [37], i.e. diabetes, high blood pressure, a heart condition, migraine, epilepsy, chronic eczema, intestinal disorder, a severe back disorder, arthritis, multiple sclerosis, a thyroid disorder, chronic bronchitis, asthma, nose allergy (such as hay fever). Women were asked whether in the past three months they had one or more infectious/inflammatory conditions, i.e. fever, flu, sore throat or throat infection, runny nose or cold, sinusitis, ear infection, pneumonia, eye infection, cold sore, mouth infection, rash, dermatitis, fungus infection of skin or feet, warts, shingles, diarrhea or enteritis, cystitis or pyelitis and jaundice. An open question followed by asking about other infectious or inflammatory condition not mentioned. Women were asked whether they had one or more of the 10 uro-genital conditions/symptoms in the past three months, i.e. urination/urethra: frequent need to urinate, pain, burning feeling, itching; vagina: discharge, burning feeling, itching; bleeding after sexual intercourse; non-painful ulceration of urethra or vagina; enlarged lymph glands in groin. We summed up the presence of chronic non-infectious conditions, infectious/inflammatory conditions or uro-genital conditions/symptoms respectively and categorized the results into three categories: none condition/symptom, one condition/symptom, two or more conditions/symptoms. Frequency of ‘sleep badly’ and ‘headache’ were measured in the same way with the measurement of nausea, vomiting and fatigue, and were dichotomized as ‘yes’ or ‘no’. Anxiety and depression were measured with two questions: “Have you ever had a period in which you were anxious or worried (for at least two consecutive weeks) and “Have you ever had a period in which you felt very down or depressed (for at least two consecutive weeks).

### Statistical analysis

Descriptive analysis was applied to characterize the study population. Differences of mean scores in physical component summary and mental component summary among subgroups were compared using one-way ANOVA. Correlations between symptoms were assessed. The Spearman correlation coefficient between nausea and vomiting was 0.50 (p<0.01); the coefficient between nausea and fatigue was 0.32 (p<0.01); the coefficient between vomiting and fatigue was 0.14 (p<0.01). Cohen’s effect sizes (*d*) were calculated by dividing the difference in mean scores among subgroups by largest SD and interpreted as: 0.2≤d<0.5 small difference, 0.5≤d<0.8 moderate difference, d≥0.8 large difference (see S2 Table) [38]. Multivariate linear regression was applied to assess the independent associations between nausea, vomiting, fatigue and scores of physical component summary and mental component summary by establishing a set of models. All models included the variable gestational age at enrollment. The crude model included three variables: frequency of nausea, vomiting and fatigue. In model 1, effect estimates were additionally adjusted by demographic characteristics. In model 2, effect estimates were additionally adjusted by the lifestyle-related factors. In model 3 (full model), we additionally adjusted by indicators of health status. Multicollinearity was checked and not serious. Multiple imputations were employed to account for the missing data in covariates. The imputed covariates were ethnic background, educational level, marital status, parity, smoking, alcohol use, headache, sleep badly, anxious or worried, feeling down and depressed, chronic non-infectious conditions, infectious/inflammatory conditions and uro-genital conditions/symptoms. Five imputed datasets were generated, based on which the pooled estimates were used to report betas and their 95% confidence intervals (CIs). Imputations were based on the relationships between all variables included in this study [39]. We also applied the multivariate linear regression analyses using the non-imputed data. Differences between women who were included in the present study (n = 5079) and women who were excluded (n = 1990) were assessed using Chi-square tests, and independent-sample t tests. Sensitivity analysis was performed by splitting the population into two subgroups: less than 14 weeks of gestation and over 14 weeks of gestation, and then comparing their outcomes (see S4 Table).

All analyses were conducted with Statistical Package for Social Sciences (SPSS) version 21.0 for Windows (IBM Corp., Armonk, NY, USA). Significance differences were indicated at the level of p<0.05.

## Results

Table 1 shows the general characteristics of the study population. In this study sample, the mean maternal age was 30 years; gestational age was less than 14 weeks of gestation in 63.7% participants. The respective percentages of daily presence of nausea, vomiting and fatigue were 33.6%, 9.6% and 44.4%. The mean score of the physical component summary was 47.73 (SD 9.03) and the mean score of the mental component summary was 48.79 (SD 10.21).

**Table 1:** Characteristics of the study population (n = 5079).

Characteristics	Value*
**Maternal age(years)**	
Mean (SD)	29.98 (4.97)
<30 years	2301 (45.3)
≥30 years	2778 (54.7)
**Gestational age(weeks)**	
Mean (SD)	13.50 (2.00)
<14 weeks	3235 (63.7)
≥ 14 weeks	1844 (36.3)
**Ethnicity background**	
Dutch	2838 (56.1)
Other western	656 (13.0)
Non-western	1567 (31.0)
**Education level**	
Low	1114 (22.2)
Mid-low	1525 (30.4)
Mid-high	1062 (21.2)
High	1311 (26.2)
**Marital status**	
Married and living together	4432 (88.0)
Single	606 (12.0)
**Parity**	
Nullipara	3027 (59.7)
Multipara	2046 (40.0)
**Smoking during first trimester(% yes)**	
**Yes, until knowing pregnancy**	657(13.1)
**Yes, still doing so**	602(12.0)
**Alcohol use during first trimester(% yes)**	
**Yes, until knowing pregnancy**	1561(31.0)
**Yes, still doing so**	888(17.6)
**If yes, how many glasses did you drink?**	
**Less than 1 glass a week**	1404(57.6)
**1 to 3 glasses a week**	701(28.8)
**4–6 glasses a week**	195(8.0)
**1 glass a day**	58(2.4)
**1–3 glasses a day**	70(2.9)
**More than 3 glasses a day**	8(0.3)
**BMI**	
**Mean±SD**	24.36±4.30
**<25**	3347(65.9)
**≥25**	1732(34.1)
**Chronic non-infectious conditions**	
**None chronic condition**	2723(56.0)
**One chronic condition**	1509(31.0)
**Two or more chronic conditions**	629(12.9)
**Infectious conditions**	
**None infectious condition**	1186(23.4)
**One infectious condition**	1287(25.4)
**Two or more infectious conditions**	2591(51.2)
**Uro-genital conditions/symptoms**	
**None condition/symptom**	681(13.5)
**One condition/symptom**	1348(26.7)
**Two or more conditions/symptoms**	3027(59.9)
**Headache(if yes)**	3553 (71.2)
**Sleep badly, (if yes)**	3690 (73.6)
**Anxious or worries (if yes)**	1469 (29.3)
**Feeling down or depressed(if yes)**	1562 (31.1)
**Nausea**	
**Daily**	1708 (33.6)
**A few days per week**	1414(27.8)
**Once per week**	425(8.4)
**Less than once per week**	663(13.1)
**Never**	869 (17.1)
**Vomiting**	
**Daily**	486(9.6)
**A few days per week**	610(12.0)
**Once per week**	425(8.4)
**Less than once per week**	663(13.1)
**Never**	2876(56.6)
**Fatigue**	
**Daily**	2256(44.4)
**A few days per week**	2000(39.4)
**Once per week**	458(9.0)
**Less than once per week**	262(5.2)
**Never**	103(2.0)
**Health-related quality of life (1–100)**	
**SF-12 Physical component summary**	
**Mean(SD)**	47.73 (9.03)
**Range**	14.07–71.55
**SF-12 Mental component summary**	
**Mean(SD)**	48.79 (10.21)
**Range**	6.74–68.88

* Values are means, SD (standard deviation), and percentages for the whole study population.

High education corresponds to university degree; mid-high level corresponds to higher vocational training, Bachelor’s degree; mid-low level corresponds to more than 3 years general secondary school, intermediate vocational training; low level corresponds to no education, primary school, lower vocational training, intermediate general school, or 3 years or less general secondary school. Data was missing for ethnicity background (n = 18), education level (n = 67), marital status (n = 41), parity (n = 18), smoking during first trimester (n = 65), alcohol use during first trimester (n = 42), uro-genital conditions/symptoms (n = 23), chronic non-infectious conditions (n = 218) and infectious conditions (n = 15), headache (n = 86), sleeping badly (n = 65), being anxious or worried (n = 61), feeling down or depressed (n = 58).

Additionally, percentages of women with multiple symptoms are presented in Table 2. 42.1% of women reported the presence of three symptoms (42.1%). Only 0.9% women reported without any symptoms. The SF-12 physical component score in women with three symptoms was relatively low compared to women without any symptoms (45.60 vs. 53.74, effect size d = 0.86).

**Table 2:** Women with the presence of multiple symptoms (nausea, vomiting and fatigue) (N = 5079).

Symptom(s)	N (%)	Physical component summary		Mental component summary	
		mean (SD)	*d*	mean (SD)	*d*
with no nausea, vomiting nor fatigue					
	47 (0.9)	53.74 (7.91)	*reference*	54.15 (7.95)	*reference*
only one symptom					
nausea	20 (0.3)	53.67 (5.42)	0.01	52.19 (7.83)	0.25 ^a^
vomiting	2 (0.04)	55.83 (5.58)	0.26^a^	49.60 (6.56)	0.57 ^b^
fatigue	792 (15.6)	50.94 (7.82)	0.35^a^	51.51 (8.59)	0.31 ^a^
Only two symptoms					
Nausea and vomiting	34 (0.6)	51.94 (9.24)	0.19	51.02 (8.86)	0.35 ^a^
Nausea and fatigue	2017 (39.7)	48.44 (8.45)*	0.63^b^	49.64 (9.72)*	0.46 ^a^
Vomiting and fatigue	28 (0.6)	48.41 (8.45)*	0.63^b^	51.62 (8.63)	0.29 ^a^
Three symptoms (nausea and vomiting and fatigue)					
	2139 (42.1)	45.60 (9.46)*	0.86^c^	46.76 (10.90)*	0.68 ^b^

*d* means effect size, which is highest minus lowest mean SF-12 score divided by the largest standard deviation.

a means small difference when 0.2≤d<0.5 small difference

b means moderate difference when 0.5≤d<0.8

c means large difference when d≥0.8; for others that d was less than 0.2, we didn’t mark them in our table.

Subgroup with no nausea, vomiting nor fatigue is the reference group when we compared the difference between subgroups.

* p<0.01.

Significant differences in physical and mental component summary scores were observed between subgroups of women who had reported the ‘daily’, ‘a few days per week’, ‘once per week’, ‘less than once per week’ or ‘never’ presence of symptoms (see S1 Table).

Independent associations between nausea, vomiting, fatigue and physical and mental component summary scores are shown in Table 3.

**Table 3:** Multiple regression analyses for associations between nausea, vomiting, fatigue and SF-12 scores (N = 5079).

	SF-12 Physical Component Score				SF-12 Mental Component Score			
	Crude model	Model 1	Model 2	Model 3	Crude model	Model 1	Model 2	Model 3
	β(95%CI)	β(95%CI)	β(95%CI)	β(95%CI)	β(95%CI)	β(95%CI)	β(95%CI)	β(95%CI)
Nausea								
Never	(Ref)	(Ref)	(Ref)	(Ref)	(Ref)	(Ref)	(Ref)	(Ref)
Less than once a week	-0.21	-0.24	-0.28	0.01	-0.79	-0.89	**-1.02**	-0.80
	(-1.07, 0.65)	(-1.10, 0.11)	(-1.15, 0.59)	(-0.88, 0.89)	(-1.80, 0.22)	(-1.89, 0.11)	**(-2.02, -0.02)**	(-1.78, 0.19)
Once a week	-0.52	-0.60	-0.72	-0.41	-0.53	-0.80	-0.80	-0.12
	(-1.51, 0.46)	(-1.60, 0.39)	(-1.72, 0.28)	(-1.44, 0.61)	(-1.70, 0.63)	(-1.95, 0.36)	(-1.96, 0.36)	(-1.26, 1.02)
Few days a week	**-1.13**	**-1.25**	**-1.24**	**-0.91**	**-1.16**	**-1.40**	**-1.59**	-0.79
	**(-1.88, -0.37)**	**(-2.00, -0.49)**	**(-2.00, -0.48)**	**(-1.69, -0.12)**	**(-2.05, -0.28)**	**(-2.27, -0.52)**	**(-2.47,-0.71)**	(-1.66, 0.08)
Daily	**-3.33**	**-3.44**	**-3.38**	**-2.95**	**-2.20**	**-2.51**	**-2.85**	**-1.74**
	**(-4.13, -2.52)**	**(-4.25, -2.64)**	**(-4.19, -2.56)**	**(-3.79, -2.12)**	**(-3.14, -1.26)**	**(-3.45, -1.58)**	**(-3.79, -1.91)**	**(-2.67, -0.80)**
Vomiting								
Never	(Ref)	(Ref)	(Ref)	(Ref)	(Ref)	(Ref)	(Ref)	(Ref)
Less than once a week	-0.66	-0.60	-0.63	-0.43	**-1.18**	-0.85	-0.85	-0.56
	(-1.34, 0.02)	(-1.28, 0.08)	(-1.31, 0.06)	(-1.13, 0.27)	**(-1.97, -0.38)**	(-1.64, -0.06)	(-1.64, -0.06)	(-1.34, 0.21)
Once a week	**-2.03**	**-1.81**	**-1.78**	**-1.55**	**-1.28**	-0.92	-0.86	-1.09
	**(-3.02,-1.03)**	**(-2.82, -0.80)**	**(-2.78, -0.77)**	**(-2.58, -0.52)**	**(-2.45, -0.11)**	(-2.09, 0.24)	(-2.02, 0.30)	(-2.24, 0.06)
Few days a week	**-2.40**	**-2.09**	**-2.08**	**-1.79**	**-1.79**	-0.71	-0.67	**-0.92**
	**(-3.19, -1.62)**	**(-2.89, -1.29)**	**(-2.88, -1.27)**	**(-2.62, -0.97)**	**(-2.71, -0.87)**	(-1.64, 0.21)	(-1.60, 0.25)	**(-1.84, -0.01)**
Daily	**-2.67**	**-2.35**	**-2.29**	**-2.08**	**-4.80**	**-3.08**	**-3.01**	**-3.39**
	**(-3.58, -1.76)**	**(-3.29, -1.40)**	**(-3.25, -1.34)**	**(-3.08, -1.09)**	**(-5.86, -3.73)**	**(-4.18, -1.98)**	**(-4.11, -1.91)**	**(-4.50, -2.29)**
Fatigue								
Never	(Ref)	(Ref)	(Ref)	(Ref)	(Ref)	(Ref)	(Ref)	(Ref)
Less than once a week	0.40	-0.55	-0.53	0.33	0.64	-0.46	-0.53	1.24
	(-2.29, 1.50)	(-2.48, 1.38)	(-2.17, 1.41)	(-1.67, 2.33)	(-1.59, 2.86)	(-2.70, 1.78)	(-2.78, 1.71)	(-0.98, 3.46)
Once a week	-0.83	-1.14	-1.03	0.33	-1.05	-2.31	**-2.35**	-0.10
	(-2.61, 0.95)	(-2.96, 0.69)	(-2.87, 0.81)	(-1.57, 0.84)	(-3.15, 1.05)	(-4.42, -0.20)	**(-4.47, -0.23)**	(-2.22, 2.02)
Few days a week	**-3.73**	**-3.94**	**-3.92**	**-2.35**	**-2.25**	**-3.47**	**-3.53**	-0.75
	**(-5.47, -0.30)**	**(-5.64, -2.25)**	**(-5.63, -2.20)**	**(-4.14, -0.56)**	**(-4.19, -0.30)**	**(-5.43, -1.51)**	**(-5.50, -1.56)**	(-2.74, 1.23)
Daily	**-7.13**	**-7.44**	**-7.42**	**-5.48**	**-5.25**	**-6.36**	**-6.34**	**-2.92**
	**(-8.78, -5.47)**	**(-9.14, -5.74)**	**(-9.13, -5.70)**	**(-7.28, -3.68)**	**(-7.20, -3.30)**	**(-8.33, -4.39)**	**(-8.32, -4.36)**	**(-4.92, -0.92)**
R square	0.16	0.17	0.17	0.20	0.09	0.13	0.14	0.21

Table 3 is based on imputed dataset. Bold print indicates statistical significance (p<0.05). Values represent betas and 95% confidence intervals derived from multiple linear regression analyses.

All models were adjusted by the gestational age at measurement.

Model 1 was adjusted by demographic characteristics (i.e. maternal age, ethnicity background, education level, parity and marital status).

Model 2 was additionally adjusted by life-style related factors (i.e. smoking, alcohol use and BMI).

Model 3 was additionally adjusted by symptoms and indicators of health status, including (i.e. headache, sleep badly, feel anxious or worried, feel down or depressed, uro-genital conditions/symptoms, chronic non-infectious conditions and infectious conditions).

Regarding to physical component summary (see Table 3), women with daily presence of nausea, vomiting and fatigue had lower scores than women without these symptoms (-3.05 [-3.84, -2.26]; -2.16 [-3.08, -1.23]; -5.19 [-6.87, -3.50]). Regarding to mental component summary, women with daily presence of nausea, vomiting and fatigue had lower scores than women without these symptoms (-1.81 [-2.72, -0.96]; -3.00 [-4.03, -1.98]; -3.00 [-4.87, -1.13]). Results based on the non-imputed data are presented in S2 Table. The profile of associations is very similar to that from the imputed data.

### Non-response analyses

Compared with the participating women in the study (n = 5079), the excluded women (n = 1990) were more often with low education, non-Dutch, single and in their first pregnancy (p<0.05) and reported lower prevalence of infectious/inflammatory conditions and uro-genital conditions/symptoms (p<0.05) (see S3 Table). Given the amount of missing data on covariates, we could not conclude that the study included healthier women, or the contrary, compared with the excluded women.

## Discussion

By far the most common pregnancy-related symptom in our study population was fatigue. Many pregnant women also reported the presence of nausea and vomiting in early pregnancy. This study shows that women with daily presence of nausea, vomiting and fatigue had lower HRQOL in both the physical and mental domains than women without these symptoms.

The average physical component summary score in our study population (47.73; SD 9.03) was below the average in a normative Dutch sample of women aged 30–39 years (53.37; SD 7.09) (p<0.01) [40]. This may reflect the presence of pregnancy-related symptoms. In our study population the subgroup of women with no symptom of nausea, vomiting or fatigue reported an average score of physical component summary as 53.74 (SD 7.91), which is very similar to the normative data (p>0.05). The average mental component summary score in our study population (48.79; SD10.21) is similar to the average in a normative Dutch sample of women aged 30–39 years (48.67; SD 10.31) (p>0.05), while the subgroup of women with no symptom of nausea, vomiting nor fatigue reported an average score of mental component summary as 54.15 (SD 7.95), which is higher than the normative data (p<0.01).

In general, the impact on the physical domain is somewhat larger in comparison with the impact on the mental domain. In the present study, pregnant women with a combination of nausea, vomiting and fatigue reported a relative low HRQOL in both physical and mental component summary scales; Cohen’s effect sizes indicate large effects of these symptoms on the physical component summary scale and moderate effects on the mental component summary scale. Based on raw data, we calculated Cohen’s effect sizes (S2 Table). These show the large effect of fatigue on the physical component summary scale (d = 0.90) and moderate effects of nausea and vomiting on both physical and mental component summary scales.

Our multivariate regression analysis showed that nausea, vomiting and fatigue are each associated with HRQOL at a significant level (p<0.05). With regard to nausea and vomiting, the result patterns are consistent with those of previous studies [9, 15, 17, 18, 41]. We also found the independent association of fatigue and HRQOL, which has not been assessed in previous studies. Specifically daily presence of fatigue is associated with a relatively low score on the physical component summary score. Fatigue is highly prevalent, and is combined with nausea and/or vomiting in most of the study population in the present study. Chou et al. showed that women with nausea and vomiting were more likely to show fatigue in early pregnancy [41]. In the present study, pregnant women with a combination of nausea, vomiting and fatigue reported a relative low HRQOL in both the physical and mental domains; Cohen’s effect sizes were large and moderate, respectively.

The presence of symptoms and the impact on HRQOL may affect the ability of women in early pregnancy to cope with demands in the workplace and other daily activities. Gadsby et al. found each year around 8.6 million hours of paid employment and 5.8 million hours of housework being lost via nausea and vomiting in the United Kingdom [6]. According to the study by Vellacott et al., about 25% women with nausea and vomiting during pregnancy reported markedly impaired job efficiency [42].

Chou et al. reported that nausea, vomiting and fatigue in early pregnancy may be associated with depressive symptoms [9], which may be an explanation for the relatively low scores in the mental domain of women with these symptoms in our study. They also suggested that this association may be mediated by the level of social support [9, 41]. So, attention for organizing social support for women experiencing these symptoms might be part of future intervention approaches [41, 43].

A recent study showed that women with nausea and vomiting during pregnancy felt their distress was trivialized by the general practitioners [44]. Health care professionals should not underestimate the presence of nausea, vomiting and fatigue in early pregnancy just because that ‘morning sickness’ is common during pregnancy. This is included into the recently published Pregnancy Nausea/Vomiting Treatment Guidelines from the American College of Obstetricians and Gynecologists [11]. Evsen et al. found that almost half of women in early pregnancy did nothing at all or ‘non-evidence based’ actions to manage nausea, vomiting or fatigue [2]. Chou et al. and O’Brien et al. also indicated that only few women with nausea and vomiting seek medical treatments [7, 19]. These findings highlight the need to be aware of negative impacts of these symptoms on HRQOL by health care professionals and pregnant women as well as their families, and accordingly necessary symptom managements should be taken under the supervision of health professionals. Since fatigue is often combined with the presence of nausea and vomiting, Donna et al. suggested that controlling fatigue may be an effective approach to manage nausea and vomiting [45]. With regard to employed women, flexible work schedule including breaks in daily life and assistance from families with daily duties in the household may help to relieve fatigue [45] and may consequently help to relieve nausea and vomiting, and improve HRQOL in these women.

### Strengths and limitations

A strength of this study is the large sample size compared to earlier studies [15, 17–21, 45]. Information regarding a comprehensive set of potential confounders was available. Some limitations should be taken into account. Causation could not be evaluated with the current cross-sectional analyses. We recommend that future studies evaluate time trajectories of nausea, vomiting, fatigue and HRQOL during pregnancy. Women who were included in the present study were younger, higher educated, more often of Dutch origin and more frequently had infectious/inflammatory conditions and uro-genital conditions/symptoms than women excluded from the sample for analysis. Given the amount of missing data on the covariates in the excluded population, we could not conclude that the excluded population was healthier or more morbid. The selection bias may have occurred; for example, if the excluded women with nausea, vomiting and fatigue provided higher (or lower) HRQOL scores than the included women with these symptoms. Furthermore, the women in this study may not fully represent the general population in the Netherlands, as all of them resided in Rotterdam. We asked women to think about the frequency of their symptoms in the previous three months, while for the most SF12 items we only asked them to recall within past month. Although we included many potential confounders in the models, remaining unmeasured confounders, such as work status [17], therapeutic approaches to relieve nausea, vomiting and fatigue, could also explain associations between nausea, vomiting, fatigue and HRQOL. Regarding the measurement of covariates in the present study, we acknowledge that the anxiety and depression were not measured by either a psychometric instrument or a diagnostic interview. The questions were unspecific, which did not capture the information of severity. Misclassification may not be ruled out.

In our study, generic HRQOL was measured. For future studies, we recommend to include both generic measures of HRQOL and specific measures such as the ‘health-related quality of life for nausea and vomiting during pregnancy’ (NVPQOL) [16]. Munch et al. showed that the NVPQOL was more sensitive to measure the impact of pregnancy-related symptoms on HRQOL compared to the SF-36 [18]. Previous studies indicated that the degree of the negative impacts of nausea and vomiting may be associated with the severity of these symptoms [17, 19]. In the present study, we measured the frequency rather than the severity of the symptoms. Women’s interpretation of the question and the framing of frequencies may have influenced the results. It is controversial that women never presented with fatigue in early pregnancy. We recommend to measure the severity by symptom-specific instruments such as the Motherisk-PUQE (pregnancy-unique quantification of emesis and nausea) scoring system [46] or the Multidimensional Assessment of Fatigue (MAF) scale [47].

## Conclusion

In this population-based study, daily presence of nausea, vomiting and fatigue was strongly associated with decreased HRQOL. This confirms the importance of paying attention by health care professionals to the presence of these symptoms and the consequences for the woman in early pregnancy. Also, social and practical support from family, relatives and friends, and adaptations with regard to work in dialogue with the employer may lead to more effective management of the impact of these pregnancy-related symptoms in early pregnancy.

## Supporting Information

S1 Checklist STROBE Statement—Checklist of items that should be included in reports of cross-sectional studies. (DOC)

S1 Fig Flow chart of population for analysis in this study. (PDF)

S1 Table Univariate analysis of SF-12 scores between subgroups according to demographic characteristics, lifestyle-related factors, indicators for health status and symptoms (n = 5079). (DOCX)

S2 Table Multiple regression analyses for associations between nausea, vomiting, fatigue and SF-12 scores using non-imputed data. (DOCX)

S3 Table Non-response analyses (n = 7069). (DOCX)

S4 Table Sensitivity analysis (n = 5079). (DOCX)
