# Supplementary figures and images for: Associations between Nausea, Vomiting, Fatigue and Health-Related Quality of Life of Women in Early Pregnancy: The Generation R Study
Source: PLoS One. 2016 Nov 4;11(11):e0166133. doi: 10.1371/journal.pone.0166133 (PMC5096665; doi:10.1371/journal.pone.0166133)

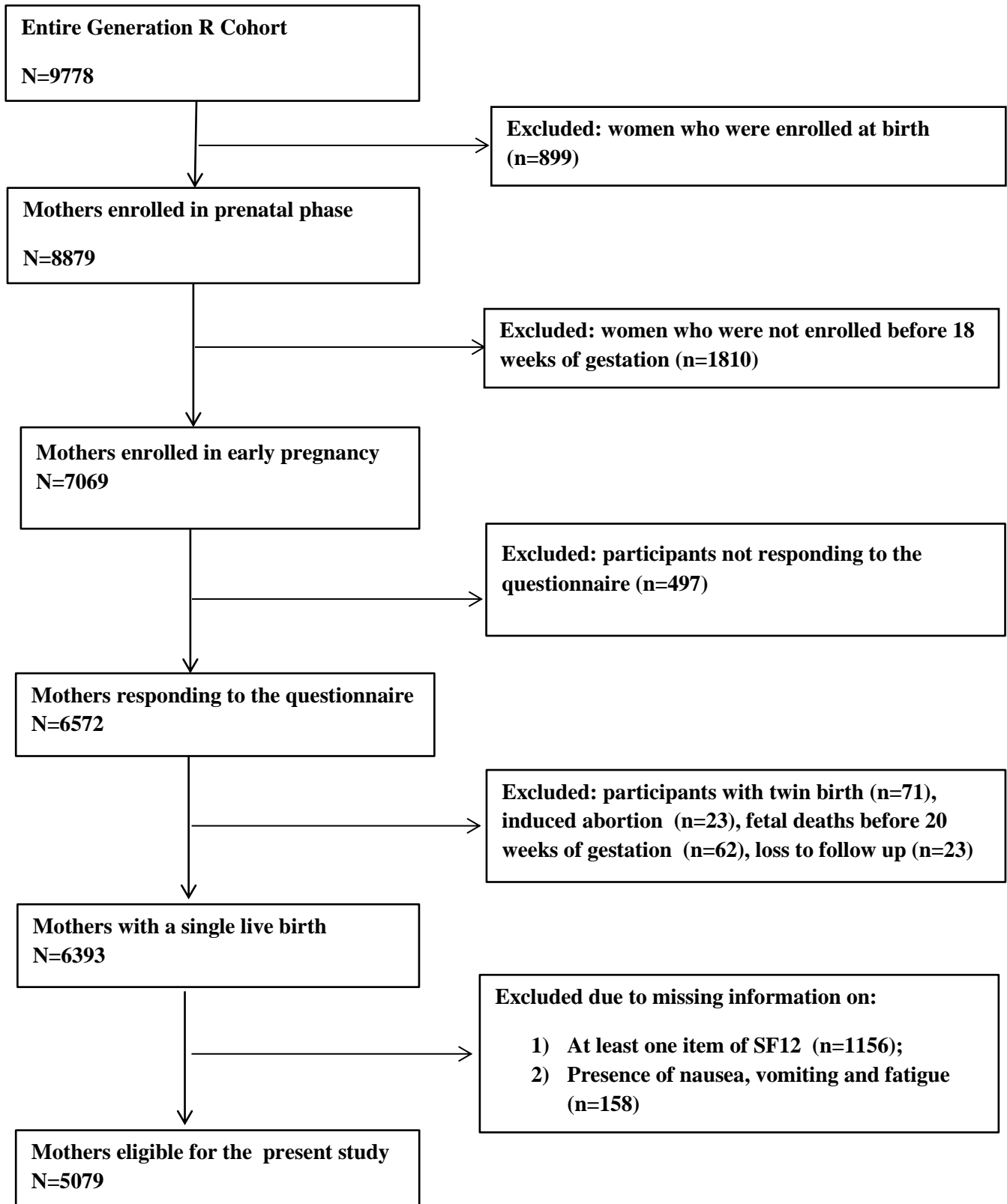

**Fig S1. Flow chart of population for analysis in this study**

Supplement: S1 Fig — (PDF) [file pone.0166133.s002.pdf]
