# Supplementary material for: Associations between Nausea, Vomiting, Fatigue and Health-Related Quality of Life of Women in Early Pregnancy: The Generation R Study
Source: PLoS One. 2016 Nov 4;11(11):e0166133. doi: 10.1371/journal.pone.0166133 (PMC5096665; doi:10.1371/journal.pone.0166133)
Supplement: S1 Table — (DOCX) [file pone.0166133.s003.docx]

| Table S1. Univariate analysis of SF-12 scores between subgroups according to demographic characteristics, lifestyle-related factors, indicators for health status and symptoms (n=5079)# | | | | | | | | |
| --- | --- | --- | --- | --- | --- | --- | --- | --- |
|  | | SF-12 Physical Component Score | | | SF-12 Mental Component Score | | | |
|  | | Mean (SD | P value | Effect size | Mean (SD | | P value | Effect size |
| Age | |  | <0.001 | 0.09 |  | | <0.001 | 0.22^a^ |
| <30 years | | 47.28 (9.23) |  |  | 47.46 (10.78) | |  |  |
| ≥30 years | | 48.10 (8.84) |  |  | 49.89 (9.58) | |  |  |
| Gestational age | |  | 0.332 | 0.03 |  | | 0.02 | 0.07 |
| <14 weeks | | 47.64 (9.00) |  |  | 49.05 (10.01) | |  |  |
| ≥14 weeks | | 47.89 (9.10) |  |  | 48.34 (10.54) | |  |  |
| Ethnicity | |  | <0.001 | 0.21^a^ |  | | <0.001 | 0.43^a^ |
| Dutch | | 48.40 (8.54) |  |  | 50.60 (8.76) | |  |  |
| Other western | | 48.12 (8.96) |  |  | 48.71 (10.61) | |  |  |
| Non-western | | 46.36 (9.72) |  |  | 45.64 (11.53) | |  |  |
| Education | |  | <0.001 | 0.19 |  | | <0.001 | 0.33^a^ |
| Low | | 47.44 (9.07) |  |  | 46.94 (10.94) | |  |  |
| Mid-low | | 47.00 (9.21) |  |  | 48.09 (10.60) | |  |  |
| Mid-high | | 47.84 (9.12) |  |  | 49.66 (9.80) | |  |  |
| High | | 48.74 (8.58) |  |  | 50.58 (9.00) | |  |  |
| Marital status | |  | 0.22 | 0.0 |  | | <0.001 | 0.4^a^ |
| Married or living together | | 47.67 (9.00) |  |  | 49.29 (9.92) | |  |  |
| No partner | | 48.15 (9.25) |  |  | 45.21 (11.49) | |  |  |
| Parity | |  | 0.39 | 0.05 |  | | 0.29 | 0.03 |
| Nullipara | | 47.65 (9.00) |  |  | 48.7 (10.2) | |  |  |
| Multipara | | 47.87 (8.90) |  |  | 49.0 (10.2) | |  |  |
| Smoking in past three months | |  | <0.001 | 0.13 |  | | <0.001 | 0.25^a^ |
| No | | 47.43 (9.04) |  |  | 49.51 (9.76) | |  |  |
| Yes | | 48.63 (8.90) |  |  | 46.70 (11.29) | |  |  |
| Alcohol use in past three months | |  | <0.001 | 0.10 |  | | <0.001 | 0.12 |
| No | | 47.59 (9.27) |  |  | 48.18 (10.52) | |  |  |
| Yes | | 48.53 (8.68) |  |  | 49.45 (9.83) | |  |  |
| BMI | |  | 0.005 | 0.08 |  | | 0.46 | 0.02 |
| <25 | | 47.98 (9.05) |  |  | 48.87 (10.22) | |  |  |
| ≥25 | | 47.24 (8.97) |  |  | 48.64 (10.20) | |  |  |
| Headache | |  | <0.001 | 0.33^a^ |  | | <0.001 | 0.27^a^ |
| Yes | | 46.86 (9.26) |  |  | 48.03 (10.50) | |  |  |
| No | | 49.91 (8.01) |  |  | 50.84 (9.08) | |  |  |
| Sleep badly | |  | <0.001 | 0.29^a^ |  | | <0.001 | 0.36^a^ |
| Yes | | 47.02 (9.20) |  |  | 47.75 (10.64) | |  |  |
| No | | 49.68 (8.24) |  |  | 51.55 (8.42) | |  |  |
| Table S1. Univariate analysis of SF-12 scores between subgroups according to demographic characteristics, lifestyle-related factors, indicators for health status and symptoms (n=5079) (continued) # | | | | | | | | |
|  | |  |  |  | |  |  |  |
|  | | SF-12 Physical Component Score | | | | SF-12 Mental Component Score | | |
|  | | Mean (SD | P value | Effect size | | Mean (SD | P value | Effect size |
| Anxious or worried | |  | <0.001 | 0.23^a^ |  | | <0.001 | 0.48^a^ |
| Yes | | 46.13 (9.73) |  |  | 44.73 (11.95) | |  |  |
| No | | 48.40 (8.65) |  |  | 50.49 (8.86) | |  |  |
| Feel down or depressed | |  | <0.001 | 0.24^a^ |  | | <0.001 | 0.52^b^ |
| Yes | | 46.12 (9.71) |  |  | 44.50 (11.95) | |  |  |
| No | | 48.48 (8.61) |  |  | 50.76 (8.65) | |  |  |
| Uro-genital symptoms | |  | <0.001 | 0.21^a^ |  | | <0.001 | 0.27^a^ |
| Yes | | 47.48 (9.07) |  |  | 48.42 (10.36) | |  |  |
| No | | 49.41 (8.52) |  |  | 51.20 (8.71) | |  |  |
| Chronic non-infectious conditions | | | <0.001 | 0.30^a^ |  | | <0.001 | 0.17 |
| Yes | 46.74 (9.22) | |  |  | 48.20 (10.56) | |  |  |
| No | 49.51 (8.35) | |  |  | 50.04 (9.25) | |  |  |
| Infectious conditions |  | | <0.001 | 0.21^a^ |  | | <0.001 | 0.19 |
| Yes | 47.30 (9.11) | |  |  | 48.33 (10.42) | |  |  |
| No | 49.22 (8.56) | |  |  | 50.27 (9.37) | |  |  |
| Nausea |  | | <0.001 | 0.71^b^ |  | | <0.001 | 0.50^b^ |
| Never | 51.02 (7.87) | |  |  | 51.65 (8.56) | |  |  |
| Less than once per week | 50.32 (7.90) | |  |  | 50.38 (9.26) | |  |  |
| Once per week | 49.53 (7.66) | |  |  | 50.28 (9.16) | |  |  |
| A few days per week | 48.12 (8.64) | |  |  | 49.18 (9.98) | |  |  |
| Daily | 44.28 (9.45) | |  |  | 46.07 (11.09) | |  |  |
| Vomiting |  | | <0.001 | 0.60^b^ |  | | <0.001 | 0.61^b^ |
| Never | 49.25 (9.03) | |  |  | 50.25 (9.43) | |  |  |
| Less than once per week | 47.83 (8.65) | |  |  | 48.44 (9.78) | |  |  |
| Once per week | 45.91 (9.60) | |  |  | 48.11 (10.51) | |  |  |
| A few days per week | 45.01 (9.41) | |  |  | 47.18 (11.13) | |  |  |
| Daily | 43.18 (10.05) | |  |  | 43.21 (11.57) | |  |  |
| Fatigue |  | | <0.001 | 0.93c |  | | <0.001 | 0.57^b^ |
| Never | 53.17 (7.90) | |  |  | 52.65 (8.24) | |  |  |
| Less than once per week | 53.00 (6.55) | |  |  | 50.08 (9.18) | |  |  |
| Once per week | 52.60 (6.86) | |  |  | 51.76 (8.55) | |  |  |
| A few days per week | 49.05 (8.10) | |  |  | 53.44 (8.08) | |  |  |
| Daily | 44.28 (9.45) | |  |  | 46.32 (11.04) | |  |  |
| Effect sizes are highest minus lowest mean SF-12 score divided by the largest standard deviation. a= small difference, | | | | | | | | |
| b=moderate difference; c= largest difference; for others that d was less than 0.2, we didn’t mark them in our table. | | | | | | | | |
| # Analysis is based on non-imputed database. | | | | | | | | |
