# Supplementary material for: Associations between Nausea, Vomiting, Fatigue and Health-Related Quality of Life of Women in Early Pregnancy: The Generation R Study
Source: PLoS One. 2016 Nov 4;11(11):e0166133. doi: 10.1371/journal.pone.0166133 (PMC5096665; doi:10.1371/journal.pone.0166133)
Supplement: S2 Table — (DOCX) [file pone.0166133.s004.docx]

Table S2. Multiple regression analyses for associations between nausea, vomiting, fatigue and SF-12 scores using non-imputed data

|  | SF-12 Physical Component Score | | | | SF-12 Mental Component Score | | | |
| --- | --- | --- | --- | --- | --- | --- | --- | --- |
|  | Crude model (N=5079) | Model 1 (N=4981) | Model 2 (N=4919) | Model 3 (N=4557) | Crude model (N=5079) | Model 1 (N=4981) | Model 2 (N=4919) | Model 3 (N=4557) |
|  | β(95%CI) | β(95%CI) | β(95%CI) | β(95%CI) | β(95%CI) | β(95%CI) | β(95%CI) | β(95%CI) |
| Nausea |  |  |  |  |  |  |  |  |
| Never | (Ref) | (Ref) | (Ref) | (Ref) | (Ref) | (Ref) | (Ref) | (Ref) |
| Less than once a week | -0.21  (-1.07, 0.65) | -0.24  (-1.10, 0.62) | -0.26  (-1.12, 0.61) | 0.04  (-0.84, 0.92) | -0.79  (-1.80, 0.22) | -0.89  (-1.89, 0.11) | **-1.06**  **(-2.05, -0.06)** | -0.83 (-1.81, 0.15) |
| Once a week | -0.52  (-1.51, 0.46) | -0.60  (-1.60, 0.39) | -0.70  (-1.70, 0.30) | -0.38  (-1.40, 0.65) | -0.53  (-1.70, 0.63) | -0.80  (-1.95, 0.36) | -0.84  (-2.01, 0.31) | -0.16  (-1.30, 0.97) |
| Few days a week | **-1.13**  **(-1.88, -0.37)** | **-1.25**  **(-2.01, -0.49)** | **-1.22**  **(-1.98, -0.45)** | **-0.88**  **(-1.66, -0.09)** | **-1.16**  **(-2.05, -0.28)** | **-1.40**  **(-2.27, -0.52)** | **-1.62**  **(-2.50,-0.74)** | -0.81  (-1.69, 0.06) |
| Daily | **-3.33**  **(-4.13, -2.52)** | **-3.44**  **(-4.25, -2.64)** | **-3.35**  **(-4.16, -2.53)** | **-2.92**  **(-3.76, -2.08)** | **-2.20**  **(-3.14, -1.26)** | **-2.51**  **(-3.45, -1.58)** | **-2.85**  **(-3.84, -1.95)** | **-1.77**  **(-2.70, -0.84)** |
| Vomiting |  |  |  |  |  |  |  |  |
| Never | (Ref) | (Ref) | (Ref) | (Ref) | (Ref) | (Ref) | (Ref) | (Ref) |
| Less than once a week | -0.66  (-1.34, 0.02) | -0.60  (-1.28, 0.08) | -0.64  (-1.32, 0.05) | -0.43  (-1.13, 0.27) | **-1.18**  **(-1.97, -0.38)** | **-0.85**  **(-1.64, -0.06)** | **-0.84**  **(-1.62, -0.05)** | -0.56  (-1.34, 0.22) |
| Once a week | **-2.03**  **(-3.02,-1.03)** | **-1.81**  **(-2.82, -0.80)** | **-1.78**  **(-2.79, -0.77)** | **-1.55**  **(-2.58, -0.52)** | **-1.28**  **(-2.45, -0.11)** | -0.92  (-2.09, 0.24) | -0.87  (-2.03, 0.30) | -1.09  (-2.23, 0.05) |
| Few days a week | **-2.40**  **(-3.19, -1.62)** | **-2.09**  **(-2.89, -1.29)** | **-2.07**  **(-2.88, -1.27)** | **-1.79**  **(-2.61, -0.96)** | **-1.79**  **(-2.71, -0.87)** | -0.71  (-1.64, 0.21) | -0.68  (-1.60, 0.25) | **-0.93**  **(-1.85, -0.02)** |
| Daily | **-2.67**  **(-3.58, -1.76)** | **-2.35**  **(-3.29, -1.40)** | **-2.29**  **(-3.24, -1.34)** | **-2.08**  **(-3.08, -1.08)** | **-4.80**  **(-5.86, -3.73)** | **-3.08**  **(-4.18, -1.98)** | **-3.02**  **(-4.12, -1.92)** | **-3.41**  **(-4.51, -2.30)** |
| Fatigue |  |  |  |  |  |  |  |  |
| Never | (Ref) | (Ref) | (Ref) | (Ref) | (Ref) | (Ref) | (Ref) | (Ref) |
| Less than once a week | 0.40  (-2.29, 1.50) | -0.55  (-2.48, 1.38) | -0.56  (-2.50, 1.38) | 0.31  (-1.68, 2.31) | 0.64  (-1.59, 2.86) | -0.46  (-2.70, 1.78) | -0.46  (-2.71, 1.78) | 1.28  (-0.94, 3.51) |
| Once a week | -0.83  (-2.61, 0.95) | -1.14  (-2.96, 0.69) | -1.07  (-2.90, 0.77) | 0.30  (-1.61, 2.21) | -1.05  (-3.15, 1.05) | **-2.31**  **(-4.42, -0.20)** | **-2.29**  **(-4.47, -0.34)** | -0.05  (-2.17, 2.07) |
| Few days a week | **-3.73**  **(-5.47, -0.30)** | **-3.94**  **(-5.64, -2.25)** | **-3.92**  **(-5.63, -2.21)** | **-2.34**  **(-4.13, -0.55)** | **-2.25**  **(-4.19, -0.30)** | **-3.47**  **(-5.43, -1.51)** | **-3.52**  **(-5.50, -1.55)** | -0.76  (-2.74, 1.23) |
| Daily | **-7.13**  **(-8.78, -5.47)** | **-7.44**  **(-9.14, -5.74)** | **-7.42**  **(-9.14, -5.71)** | **-5.47**  **(-7.27, -3.67)** | **-5.25**  **(-7.20, -3.30)** | **-6.36**  **(-8.33, -4.39)** | **-6.34**  **(-8.31, -4.35)** | **-2.92**  **(-4.92, -0.92)** |
| R square | 0.16 | 0.17 | 0.17 | 0.20 | 0.09 | 0.13 | 0.14 | 0.21 |

Table is based on non-imputed dataset. Bold print indicates statistical significance (p<0,05). Values represent betas (95% confidence intervals) and R squares derived from multiple linear regression analyses.

All models are adjusted by gestational age at measurement.

Model 1 was adjusted by demographic characteristics (i.e. maternal age, ethnicity background, education level, parity and marital status); Model 2 was additionally adjusted by life-style related factors (i.e. smoking, alcohol use and BMI); Model 3 was additionally adjusted by symptoms and indicators of health status, including(i.e. headache, sleep badly, feel anxious or worried, feel down or depressed, uro-genital symptoms, chronic non-infectious conditions and infectious conditions).
