# Supplementary material for: Associations between Nausea, Vomiting, Fatigue and Health-Related Quality of Life of Women in Early Pregnancy: The Generation R Study
Source: PLoS One. 2016 Nov 4;11(11):e0166133. doi: 10.1371/journal.pone.0166133 (PMC5096665; doi:10.1371/journal.pone.0166133)
Supplement: S3 Table — (DOCX) [file pone.0166133.s005.docx]

Table S3. Non-response analyses (n=7069)

| Characteristics | population for analysis (n=5079)* | Excluded population  (n=1990)** | P value^&^ |
| --- | --- | --- | --- |
| Maternal age(years) |  |  |  |
| Mean(SD) | 29.98 (4.97) | 29.13 (5.44) | <0.001 |
| Range | 15.27-46.34 | 15.50-43.98 |  |
| <30 years | 2301 (45.3) | 1053 (52.9) | <0.001 |
| ≥30 years | 2778 (54.7) | 937 (47.1) |  |
| Gestational age(weeks) |  |  |  |
| Mean(SD) | 13.21(10.50-17.21) (2.00) | 13.36(10.36-17.36) | 0.027 |
| Range | 4.50-17.98 | 5.70-17.93 |  |
| <14 weeks | 3235 (63.7) | 1191 (59.8) | 0.003 |
| ≥ 14 weeks | 1844 (36.3) | 799 (40.2) |  |
| Ethnicity background |  |  | <0.001 |
| Dutch | 2838 (56.1) | 652 (38.3) |  |
| Other western | 656 (13.0) | 163 (9.6) |  |
| Non-western | 1567 (31.0) | 887 (52.1) |  |
| Education level |  |  | <0.001 |
| Low | 1114 (22.2) | 516 (33.8) |  |
| Mid-low | 1525 (30.4) | 482 (31.6) |  |
| Mid-high | 1062 (21.2) | 264 (17.3) |  |
| High | 1311 (26.2) | 263 (17.2) |  |
| Marital status |  |  | <0.001 |
| Married and living together | 4432 (88.0) | 1200 (81.6) |  |
| Single | 606 (12.0) | 270 (18.4) |  |
| Parity |  |  | <0.001 |
| Nullipara | 2046 (51.4) | 961 (66.2) |  |
| Multipara | 3027 (48.6) | 961 (33.8) |  |
| Smoking in past three months(%) |  |  | 0.20 |
| No | 3755 (74.9) | 836 (74.0) |  |
| Yes, until knowing pregnancy | 657 (13.1) | 137 (12.1) |  |
| Yes, still doing so | 602 (12.0) | 156 (13.8) |  |
| Alcohol use in past three months(%) |  |  | <0.001 |
| No | 2588 (51.4) | 756 (38.0) |  |
| Yes, until knowing pregnancy | 1561 (31.0) | 265 (23.2) |  |
| Yes, still doing so | 888 (17.6) | 121 (10.6) |  |
| BMI |  |  |  |
| Mean±SD | 24.36 (4.30) | 25.17 (4.80) | <0.001 |
| Range | 15.60-50.61 | 15.70-49.10 |  |
| <25 | 3347 (65.9) | 1125 (56.5) | <0.001 |
| ≥25 | 1732 (34.1) | 865 (43.5) |  |
| Uro-genital symptoms |  |  | <0.001 |
| None symptom | 681 (13.5) | 555 (37.6) |  |
| One symptom | 1348 (26.7) | 292 (19.8) |  |
| Two or more symptoms | 3027 (59.9) | 630 (42.7) |  |
| Chronic non-infectious conditions |  |  | 0.27 |
| None condition | 2603 (55.6) | 477 (52.7) |  |
| One condition | 1276 (27.3) | 260 (28.7) |  |
| Two or more conditions | 802 (17.1) | 168 (18.6) |  |
| Infectious/inflammatory conditions |  |  | <0.001 |
| None condition | 1186 (23.4) | 685 (46.2) |  |
| One condition | 1287 (25.4) | 253 (17.0) |  |
| Two or more conditions | 2591 (51.2) | 546 (36.8) |  |
| Headache(if yes) | 3553 (71.2) | 798 (78.8) | <0.001 |

Table S3. Non-response analyses (n=7069) (continued)

| Characteristics | population for analysis (n=5079)* | Excluded population  (n=1990)** | P value^&^ |
| --- | --- | --- | --- |
| Sleep badly, (if yes) | 3690 (73.6) | 768 (76.6) | 0.05 |
| Anxious or worries (if yes) | 1469 (29.3) | 312 (28.2) | 0.48 |
| Feeling down or depressed(if yes) | 1562 (31.1) | 337 (30.3) | 0.61 |

Values are absolute numbers (percentages) for categorical variables or means (standard deviation) for continues variables. * Data was missing for ethnicity background (n=18), education level (n=67), marital status (n=41), parity (n=18), smoking during first trimester (n=65), alcohol use during first trimester (n=42), uro-genital symptoms (n=23), chronic non-infectious conditions (n=398) and infectious/ inflammatory conditions (n=15), headache (n=86), sleep badly (n=65), anxious or worried (n=61), feeling down or depressed (n=58). **Data was missing for ethnicity background (n=288), education level (n=465), marital status (n=520), parity (n=69), smoking in past three months(n=861), alcohol use in past three months (n=848), headache (n=977), sleep badly (n=987), anxious or worried (n=884), feeling down or depressed (n=879), uro-genital symptoms (n=513), chronic non-infectious conditions (n=1085) and infectious/ inflammatory conditions (n=506). ^&^Independent-sample t tests for continuous variables and Chi-square tests for categorical variables.
