# Supplementary material for: Associations between Nausea, Vomiting, Fatigue and Health-Related Quality of Life of Women in Early Pregnancy: The Generation R Study
Source: PLoS One. 2016 Nov 4;11(11):e0166133. doi: 10.1371/journal.pone.0166133 (PMC5096665; doi:10.1371/journal.pone.0166133)
Supplement: S4 Table — (DOCX) [file pone.0166133.s006.docx]

Table S4. Sensitivity analysis (n=5079)

|  | SF-12 Physical Component Score | | SF-12 Mental Component Score | |
| --- | --- | --- | --- | --- |
|  | <14 weeks | ≥14 weeks | <14 weeks | ≥14 weeks |
| Nausea |  |  |  |  |
| Never | (Ref) | (Ref) | (Ref) | (Ref) |
| Less than once a week | 0.17 (-0.94, 1.28) | -0.49 (-1.98, 1.01) | -1.22 (-2.44, 0.01) | -0.10 (-1.79, 1.59) |
| Once a week | -0.05 (-1.36, 1.25) | -0.98 (-2.65, 0.68) | -0.50 (-1.93, 0.94) | 0.30 (-1.58, 2.19) |
| Few days a week | -0.90 (-1.88, 0.08) | -0.93 (-2.21, 0.39) | **-1.12 (-2.20, -0.04)** | -0.38 (-1.88, 1.12) |
| Daily | **-2.97 (-4.03, -1.90)** | **-2.83 (-4.21, -1.44)** | **-2.11 (-3.28, -0.94)** | -1.31 (-2.87, 0.26) |
| Vomiting |  |  |  |  |
| Never | (Ref) | (Ref) | (Ref) | (Ref) |
| Less than once a week | -0.38 (-1.24, 0.48) | -0.44 (-1.64, 0.77) | -0.41 (-1.36, 0.54) | -0.97 (-2.24, 0.39) |
| Once a week | **-1.99 (-3.24, -0.74)** | -0.87 (-2.70, 0.97) | -1.02 (-2.40, 0.36) | -1.24 (-3.31, 0.84) |
| Few days a week | **-1.87 (-2.92, -0.82)** | **-1.59 (-2.94, -0.23)** | **-1.25 (-2.41, -0.10)** | -0.49 (-2.02, 1.04) |
| Daily | **-1.78 (-3.05, -0.53)** | **-2.45 (-4.06, -0.84)** | **-3.78 (-5.17, -2.38)** | **-2.92 (-4.74, -1.09)** |
| Fatigue |  |  |  |  |
| Never | (Ref) | (Ref) | (Ref) | (Ref) |
| Less than once a week | 1.96 (-0.56, 4.47) | -1.77 (-5.09, 1.56) | 1.11 (-1.66, 3.88) | 1.63 (-2.14, 5.39) |
| Once a week | 1.70 (-0.71, 4.10) | -1.90 (-5.04, 1.25) | -0.17 (-2.82, 2.48) | -0.04 (-3.60, 3.58) |
| Few days a week | -0.63 (-2.88, 1.62) | **-5.01 (-7.97, -2.05)** | -1.08 (-3.56, 1.41) | -0.28 (-3.62, 3.07) |
| Daily | **-3.76 (-6.04, -1.49)** | **-8.09 (-11.07, -5.11)** | **-3.29 (-5.80, -0.79)** | -2.28 (-5.64, 1.09) |
| R square | 0.21 | 0.20 | 0.22 | 0.23 |

Data was based on the non-imputed dataset. Bold print indicates statistical significance (p<0,05). Values represent betas (95% confidence intervals) and R squares derived from multiple linear regression analyses.

The results are based on full models adjusted by all covariates (i.e. maternal age, gestational age, ethnicity background, education level, parity and marital status, smoking, alcohol use and BMI, headache, sleep badly, feel anxious or worried, feel down or depressed, uro-genital symptoms, chronic non-infectious conditions and infectious conditions).
